# Investigation of heat stress responses and adaptation mechanisms by integrative metabolome and transcriptome analysis in tea plants (*Camellia sinensis*)

**DOI:** 10.1038/s41598-024-60411-0

**Published:** 2024-05-01

**Authors:** Feiyi Huang, Yu Lei, Jihua Duan, Yankai Kang, Yi Luo, Ding Ding, Yingyu Chen, Saijun Li

**Affiliations:** Tea Research Institute in Hunan Academy of Agricultural Sciences/National Small and Medium Leaf Tea Plant Germplasm Resource Nursery (Changsha)/National Centre for Tea Improvement, Hunan Branch, Changsha, 410125 China

**Keywords:** *Camellia sinensis*, Heat stress, Molecular chaperone, Flavonoids, Photosynthesis, Metabolomics, Plant molecular biology, Drought

## Abstract

Extreme high temperature has deleterious impact on the yield and quality of tea production, which has aroused the attention of growers and breeders. However, the mechanisms by which tea plant varieties respond to extreme environmental heat is not clear. In this study, we analyzed physiological indices, metabolites and transcriptome differences in three different heat-tolerant tea plant F1 hybrid progenies. Results showed that the antioxidant enzyme activity, proline, and malondialdehyde were significantly decreased in heat-sensitive ‘FWS’ variety, and the accumulation of reactive oxygen molecules such as H_2_O_2_ and O_2_^−^ was remarkably increased during heat stress. Metabolomic analysis was used to investigate the metabolite accumulation pattern of different varieties in response to heat stress. The result showed that a total of 810 metabolites were identified and more than 300 metabolites were differentially accumulated. Transcriptional profiling of three tea varieties found that such genes encoding proteins with chaperon domains were preferentially expressed in heat-tolerant varieties under heat stress, including universal stress protein (*USP32*, *USP-like*), chaperonin-like protein 2 (*CLP2*), small heat shock protein (*HSP18.1*), and late embryogenesis abundant protein (*LEA5*). Combining metabolomic with transcriptomic analyses discovered that the flavonoids biosynthesis pathway was affected by heat stress and most flavonols were up-regulated in heat-tolerant varieties, which owe to the preferential expression of key *FLS* genes controlling flavonol biosynthesis. Take together, molecular chaperons, or chaperon-like proteins, flavonols accumulation collaboratively contributed to the heat stress adaptation in tea plant. The present study elucidated the differences in metabolite accumulation and gene expression patterns among three different heat-tolerant tea varieties under extreme ambient high temperatures, which helps to reveal the regulatory mechanisms of tea plant adaptation to heat stress, and provides a reference for the breeding of heat-tolerant tea plant varieties.

## Introduction

The tea plant (*Camellia sinensis*) is one of the most important economic crops and is widely cultivated in tropical and sub-tropical regions of the world^1^. The leaves of the tea plant ware usually processed into an immensely popular beverage due to its fascinating taste, flavor, aroma, and healthy properties^2^. Due to the frequent occurrence of extreme hyperthermia in recent years, tea plants are often exposed to heat stress in a natural environment, which might cause adverse effects on the growth and development of tea plants^3^. According to the records, the global temperature increase has accelerated significantly from 0.19 °C per decade to 0.25 °C per decade, which is a faster increase than the mean annual temperature^4^. Therefore, global warming in last decades has been one of the major threats to tea production, and the yield and quality in various tea-producing areas are significantly restricted and deteriorated by the frequent extreme high-temperature^5^. However, sustainable producing of tea yields and quality requires enhanced efforts through a multi-objective approach to develop climate-resilient tea varieties.

Previous findings have suggested that heat stress adversely damaged the chloroplast and mitochondria ultrastructure, photosystem II, and membrane thermostability, which ultimately influenced the yield and quality of crops^6–8^. In tea plants, water retention, chlorophyll content, and oxidation potential were reduced, and membrane damage was increased during heat stress^2^. Heat stress primarily not only deteriorated membrane fluidity but also simultaneously served as a stress signal for triggering hormone signaling pathways, resulting in reactive oxygen species (ROS) accumulation, cytosolic calcium increasing, and heat-related gene expression^9^. One of the salient features is the production of ROS, such as superoxide anion (O_2_^−^), hydrogen peroxide (H_2_O_2_), hydroxyl radical (HO^−^), perhydroxyl radical (HO_2_^**·**^) and singlet oxygen (^1^O2) within the cell during heat stress^9,10^. ROS induce oxidative damage by accelerating lipid peroxidation, protein degradation, and enzyme inactivation and altering nucleic acid stabilization, resulting in plant cell viability^11^. Therefore, plants have evolved complex molecular and biochemical mechanisms to eliminate ROS. The plants can recruit antioxidant enzymes, such as peroxidase (POD), ascorbate peroxidase (APX), catalase (CAT), and superoxide dismutase (SOD), to detoxify superabundant reactive oxygen species (ROS) and enhance heat stress^12–14^. Additionally, the key enzymes involved in the synthesis and decomposition of various compounds are also influenced by heat stress, such as amino acids, proteins, soluble sugars, and lipids, leading to the alteration of the accumulation of secondary metabolites^15^. The accumulation of some beneficial substances can also be affected during exposure to continuous high temperatures. The theanine biosynthetic genes were suppressed by heat stress, resulting in decreased theanine concentration, which is the most abundant free amino acid in tea leaves that contributes to the unique umami flavor of green tea^3,16^. However, the accumulation pattern of metabolites under continuous heat stress has not been investigated in detail yet in tea plants.

The molecular mechanism of plant response to heat stress has been extensively investigated in *Arabidopsis*, and the regulatory network with heat shock factor (HSF) as the core has been deciphered in detail^17^. In the tea plant, 25 HFS family genes were identified, and *CsHsfA2* was proven to improve thermotolerance in transgenic yeast^18,19^. Among HSF family genes, the *HSFA1* group served as the master transcriptional activator, triggering the downstream expression of other genes associated with heat stress, including *DREB2A*, *HSFA2*, *HSFA7*, *HSFBs*, and *MBF1C*^14^. In addition, heat shock proteins (HSPs) were dynamically regulated by the activation of HSFs for the tolerance and acquisition of thermotolerance in plants^20,21^. Recently, multiple findings have improved the critical roles of HSPs in sensing and signaling heat stress^22^. The molecular chaperone HSP70 and HSP90 were proven to inhibit *HSFA1* transactivation activity^23^. A total of 47 HSP family genes were isolated from the tea plant genome^24^. In addition, some small heat shock proteins (sHSP) also function as molecular chaperones and play crucial roles in plants. Such as chloroplast CsHSP24.6 was induced by heat stress and interacted with target gene *CspTAC5* to enhance the heat stress in tea plants^25^. CsHSP17.2 also acts as a molecular chaperone to improve heat tolerance by enhancing photochemical efficiency and protein synthesis, scavenging ROS, and inducing the expression of heat stress-related genes^26^. In addition, a number of universal stress proteins (USPs), which were found in diverse sources, including archaea, bacteria, plants, and metazoans, served as molecular chaperones to participate in cellular responses to heat stress^27^. In *Arabidopsis*, previous findings suggested that the chaperone function of *AtUSP* was significantly increased by heat treatment, and over-expressed *AtUSP* showed a strong resistance to heat shock and oxidative stress^28^. However, limited information on tea plant response to heat stress was also considered.

Therefore, the current study intends to unravel the metabolism accumulation and transcript expression pattern of tea varieties with different heat resistance by correlating the physiological, metabolomic, and transcriptomic analyses under long-term high temperature. The phenotypic observations and physiological detections based on the thermotolerance response were performed. The study found multiple metabolic pathways, including amino acid metabolism, photosynthesis, and the flavonoid biosynthesis pathway, were significantly influenced among different heat-tolerant tea varieties. In addition, it was noted that the sHSP family genes, or molecular chaperon-like genes, were remarkably up-regulated in heat-tolerant tea varieties during long-term high temperature. This finding was conducive to perform a deeper understanding of chaperons-mediated regulation of heat stress responses in tea plants.

## Results

### Heat stress tolerance differences among tea varieties

**Figure 1:**
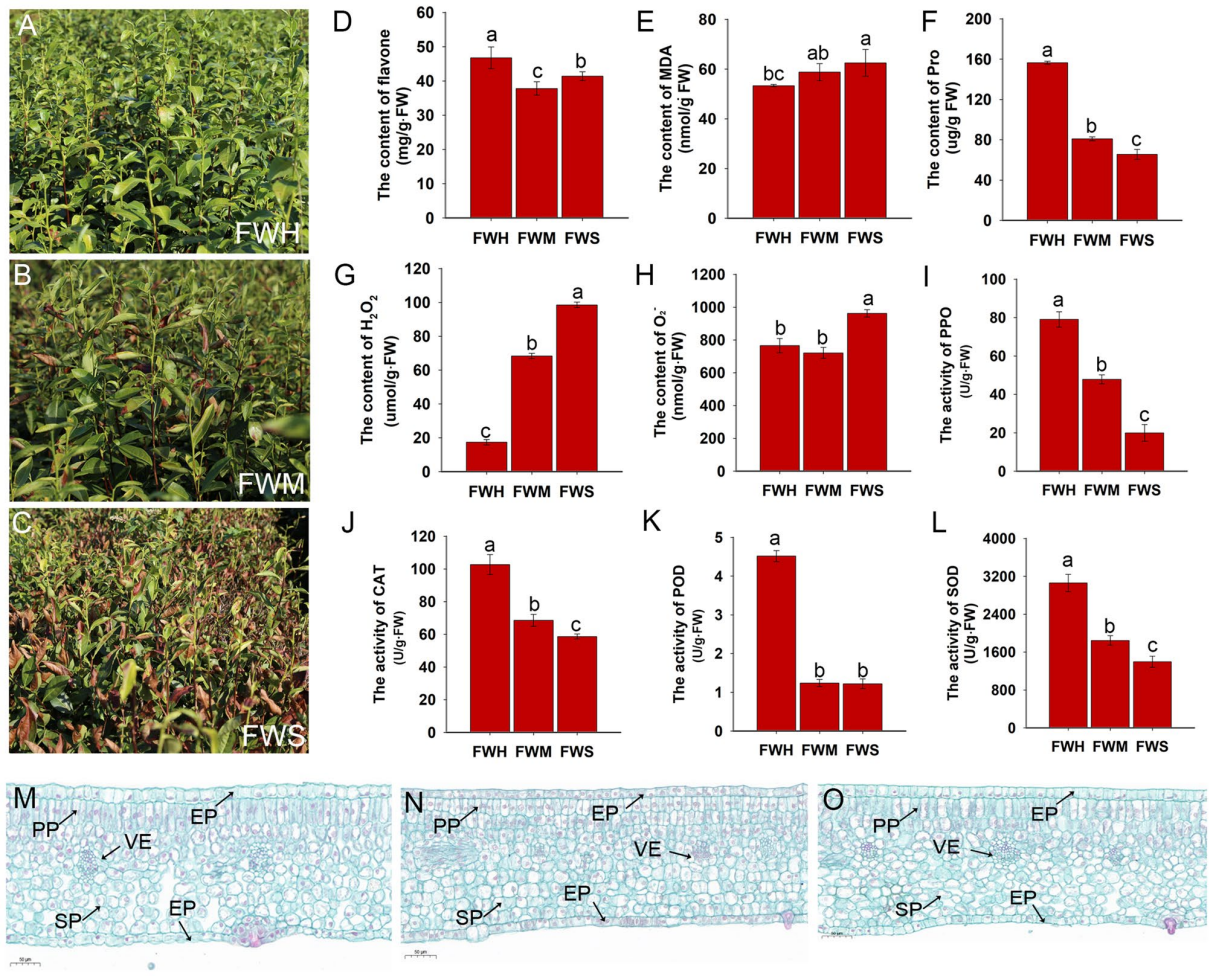
Phenotypic and physiological traits of different varieties under heat stress. (**A**–**C**) Phenotypic trats of ‘FWH’, ‘FWM’, and ‘FWS’ under heat stress. (**D**–**L**) The effects of heat stress on physiological traits of tea plants; (**D**–**H**) The content of flavone, MDA, Pro, H_2_O_2_, and O_2_^−^; (**I**–**L**) The activity of PPO, CAT, POD, and SOD; (**M**–**O**) The leaf structure of ‘FWH’, ‘FWM’, and ‘FWS’ under heat stress. Paraffin section was used to performed and the tissue was stained by safranin and fast green. *SP* sponge tissue, *PP* palisade tissue, *EP* epidermal cell layer, *VE* leaf vein.

In our previous work, a heat -sensitive variety ‘Wuniuzao (WNZ)’ and a heat-tolerant variety ‘Fudingdabaicha (FD)’ were used as female and male parents, respectively, to generate F1 hybrid progenies, named ‘FWH’, ‘FWM’, and ‘FWS’ in this work. We found that the F1 offspring that were cultivated in the same tea plantation had a different heat-tolerant phenotype under the natural high temperature. The young leaves of ‘FWH’ were aways green and hardly damaged by the heat attack (Fig. 1A). And some of the young leaves have shriveled and died in ‘FWM’ offspring (Fig. 1B). What's more serious is that the leaves ‘FWS’ were severely shriveled and died under the heat attack (Fig. 1C). The content of flavone was the highest in ‘FWH’ (46.8 ± 3.2 mg/g FW) and significantly reduced in ‘FWM’ (37.8 ± 1.9 mg/g FW) varieties (Fig. 1D). The content of malondialdehyde (MDA) induced by lipid peroxidation of polyunsaturated fatty acids was significantly increased in ‘FWM’ (58.8 ± 3.4 nmol/g FW) and ‘FWS’ (62.5 ± 5.3 nmol/g FW) variety under the heat attack (Fig. 1E), and the content of proline (Pro) was the highest in ‘FWM’ (156.5 ± 1.5 μg/g FW) (Fig. 1F). The content of H_2_O_2_ was significantly increased in ‘FWM’ (68.4 ± 1.5 μmol/g FW) and ‘FWS’ (98.5 ± 1.7 μmol/g FW) after heat stress (Fig. 1G) while the content of O_2_^−^ was the highest in variety ‘FWS’ (962.4 ± 22.4 nmol/g FW) (Fig. 1H). The activity of polyphenol oxidase (PPO) which catalyzes the oxidation of polyphenols to quinones was the highest in ‘FWH’ (79.0 ± 4.0 U/g FW) and the lowest in ‘FWS’ (19.9 ± 4.3 U/g FW) (Fig. 1I). The main reactive oxygen species (ROS) scavenging enzymes, such as catalase (CAT), superoxide dismutase (SOD), and peroxidase (POD), were also measured, and it was found that the activity of CAT, SOD, and POD was significantly increased in ‘FWH’ (Fig. 1J–L). The anatomical traits were compared among three varieties under heat attack. As shown in Fig. 1M–O, significant differences were not observed through the section structure, including epidermal cell layer (EP), palisade tissue (PP), sponge tissue (SP), and leaf vein (VE). These results indicate that the heat-tolerant variety has strong active oxygen scavenging ability, and the leaf structure obviously fails to vary after heat stress among different tea varieties.

### Metabolomic analysis of tea plant after heat stress

**Figure 2:**
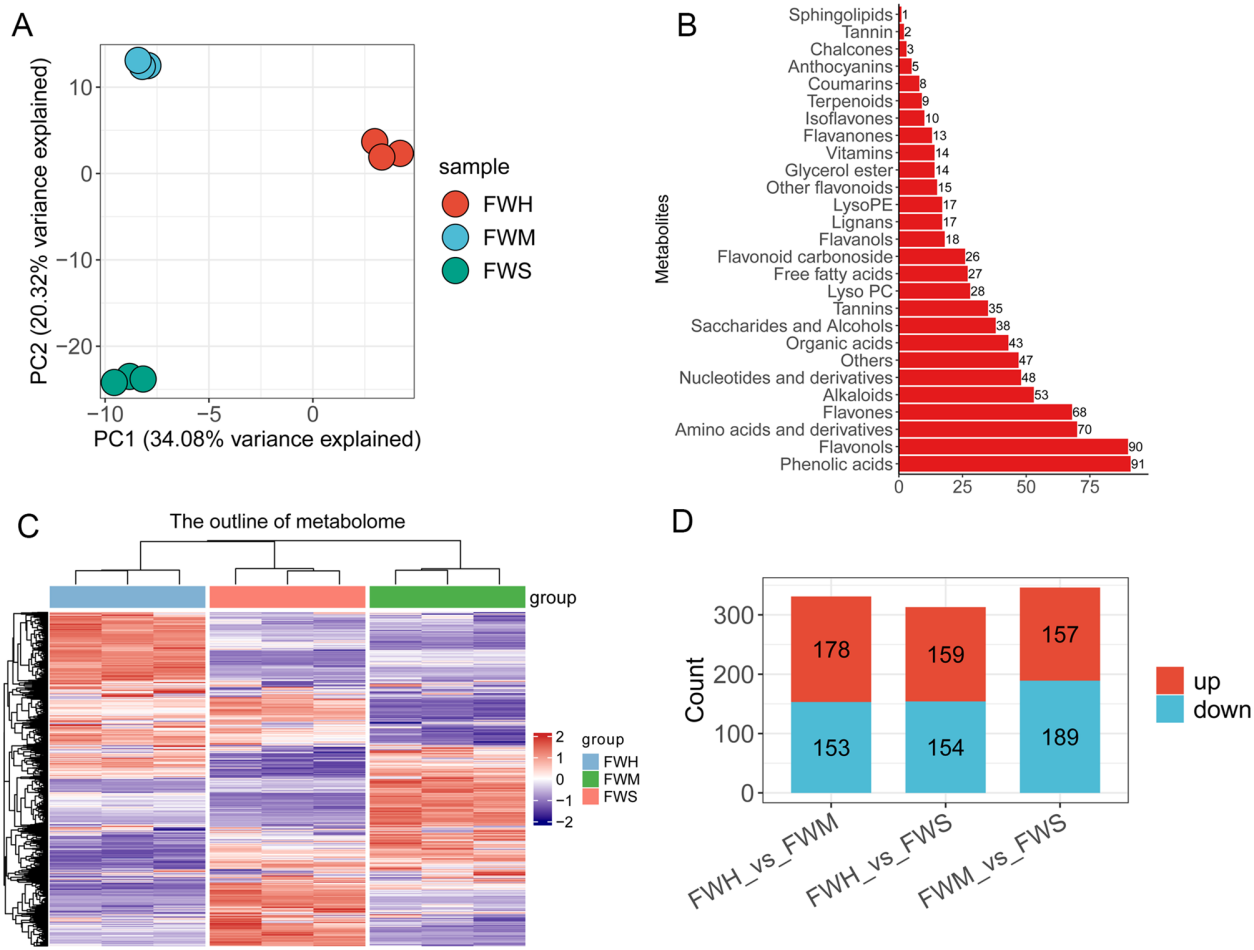
The outline of accumulated metabolites of tea varieties after heat stress. (**A**) Principal component analysis (PCA) score plots among nine samples (three varieties); (**B**) The metabolites classified through the metabolomic analysis; (**C**) Hierarchical clustering analysis of all metabolites identified from the nine samples; (**D**) Comparison of up- and down-regulated metabolites among different tea varieties after heat stress.

To comprehensively investigate the metabolite differences, the UPLC-ESI–MS/MS system was used. A total of 810 metabolites were identified from nine samples (Supplementary Table S1). Principal component analysis (PCA) was used to analyze the data, and we plotted the 9 samples in PCA diagrams using different colors (Fig. 2A). PC1 and PC2 explained more than 50% of the total variation and separated the samples without any overlap, indicating that the significant dissimilarity of metabolites among tea varieties after heat stress (Fig. 2A). In this work, all metabolites were classified into 27 categories, and the most enriched metabolites were phenolic acids (91 metabolites), followed by flavonols (90 metabolites), amino acids and derivatives (70 metabolites), flavones (68 metabolites), and alkaloids (53 metabolites) (Fig. 2B). As shown in Fig. 2C, the cluster analysis in metabolite from three tea varieties exhibited a clear accumulation pattern, indicating that a high degree of diversity of metabolites accumulation was induced by heat stress. The differentially accumulated metabolites were compared. The group FWH_vs_FWS produced the lowest number of differential metabolites (n = 313; 159 up-regulated and 154 down-regulated), while the groups FWH_vs_FWM and FWM_vs_FWS had similar differential metabolites (331 and 346 metabolites) (Fig. 2D, Supplementary Table S2). These results suggested that the three tea varieties showed significantly different metabolite accumulation patterns after high temperature stress.

### KEGG enrichment of differential metabolites

**Figure 3:**
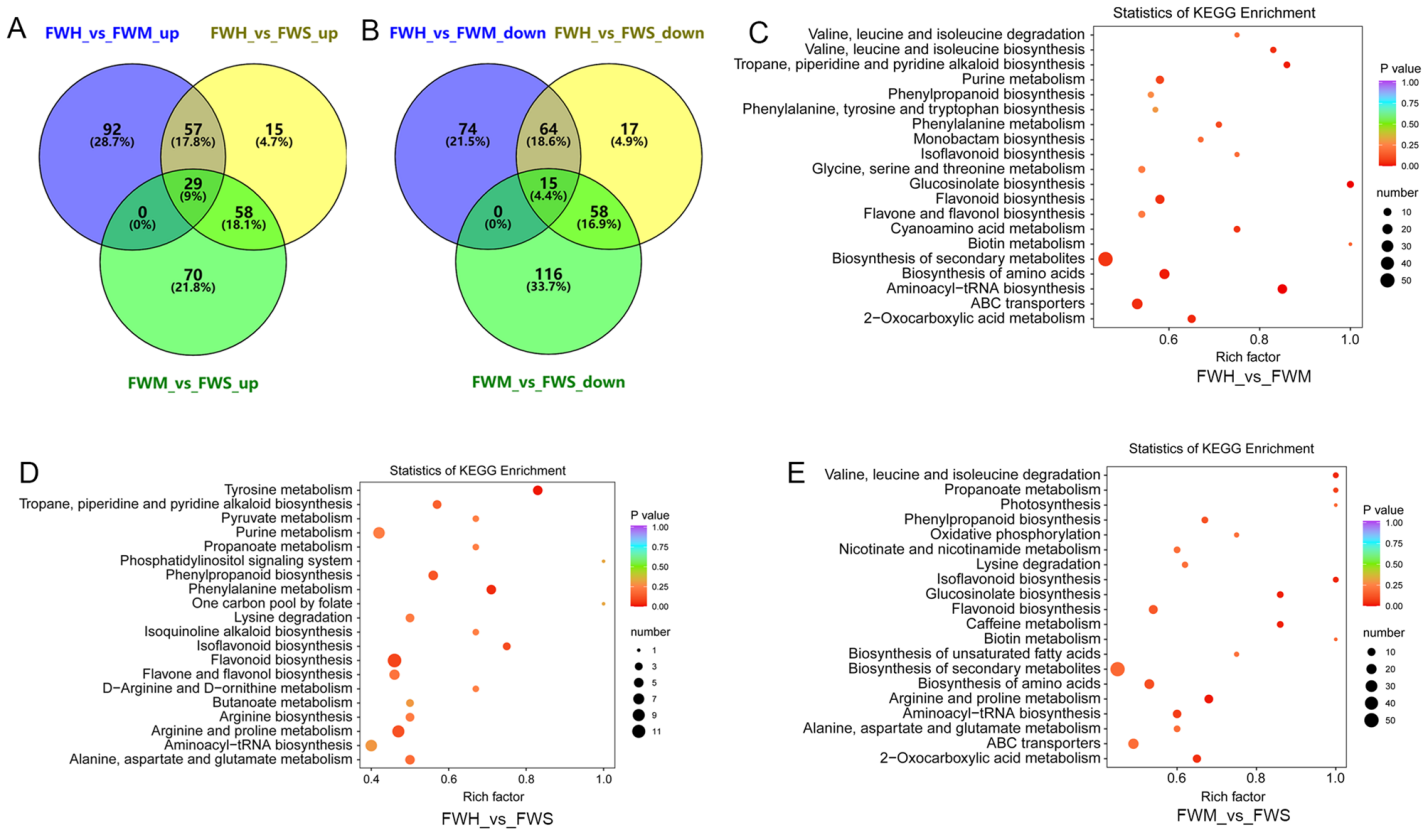
Identification of differential metabolites and KEGG enrichment in three groups. (**A**,**B**) Venn diagram of differential metabolites in three groups based on the up- and down-accumulated pattern; (**C**–**E**) The main KEGG enrichment pathway of differential metabolites in three comparison groups. Figure source: www.kegg.jp/kegg/kegg1.html.

To comprehensively investigate the metabolite accumulation differences in different tea varieties after heat stress, the differential metabolites were further analyzed. In this work, only 29 up-accumulated metabolites and 15 down-accumulated metabolites were commonly identified in all comparable groups (Fig. 3A,B). The obtained differential metabolites were further used to construct KEGG analyses against the annotated canonical pathways. 20 metabolic pathways were significantly enriched in all groups, respectively. These pathways included the biosynthesis of secondary metabolites, ABC transporters, the biosynthesis of amino acids, and flavonoid biosynthesis in FWH_vs_FWM (Fig. 3C). Notably, a substantial number of differential metabolites were enriched into d-arginine and d-ornithine metabolism, arginine biosynthesis, arginine and proline metabolism, and alanine, aspartate and glutamate metabolism in FWH_vs_FWS (Fig. 3D). Compared to FWH_vs_FWM, similar pathways were enriched in FWM_vs_FWS, including the biosynthesis of secondary metabolites, the biosynthesis of amino acids, arginine and proline metabolism, alanine, aspartate and glutamate metabolism, ABC transporters and 2-Oxocarboxylic acid metabolism (Fig. 3E).

### Transcriptomic analysis of tea plants in response to heat stress

For transcriptomic analysis among three tea varieties, high-quality total RNA of leaves under heat stress was extracted and transcribed into cDNAs. Nine cDNA libraries were further amplified and constructed. A total of 41.7–44.6 million raw reads were produced by using the Illumina^®^ HiSeq2500 platform (Supplementary Table S3). After processing raw reads, an average 6.7 G clean base with a Q30 percentage of 95.0–97.25% was obtained. The GC content percentage of 45.13–46.15% was obtained and was available for analysis (Supplementary Table S3). All the reads were successfully mapped to the reference genome with a percentage of 92.45–92.85%, and 84.73–87.58% of the reads were uniquely mapped (Supplementary Table S3). These results showed that high-quality sequencing data can be used for further analyses.

**Figure 4:**
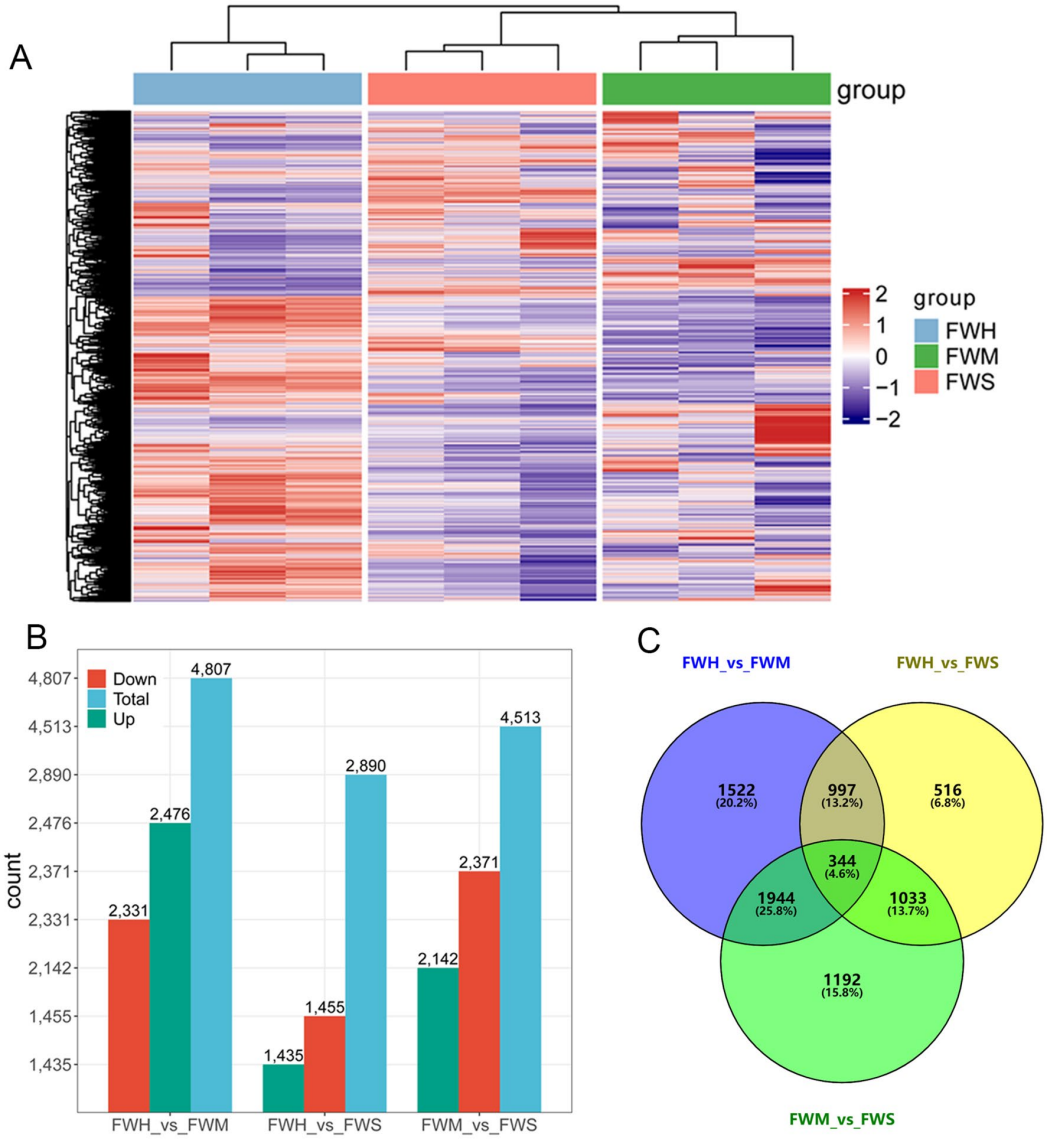
Analysis of gene expression under heat treatment in tea plants. (**A**) Hierarchical clustering of genes identified in all samples; (**B**) Number of DEGs at any two different tea varieties; (**C**) Venn diagram of DEGs commonly and uniquely expressed among the comparison in pairs.

Totally, 33, 973 genes were identified and annotated by mapping to the reference genome of ‘Shuchazao’ (*C. sinensis* var. sinensis) (Supplementary Table S4). To obviously outline the gene expression pattern among tea varieties, hierarchical cluster analysis was conducted based on the standard FPKM value for each gene. As shown in Fig. 4A, a high degree of consistency in gene expression patterns was observed among groups. Notably, compared to FWM and FWS varieties, the expression pattern was obviously different in FWH varieties, a heat-resistant variety. The differentially expressed genes (DEGs) among the varieties were determined by the fold-change of the FPKM value. In FWH_vs_FWM, 4807 DEGs were identified, of which 2476 were up-regulated, and 2331 were down-regulated. In FWH_vs_FWS, 2890 DEGs were detected, of which 1455 were up-regulated, and 1435 were down-regulated. In FWM_vs_FWS, 4513 DEGs were noted, of which 2371 were up-regulated, and 2142 were down-regulated (Fig. 4B, Supplementary Table S5). In order to identify genes that potentially contribute to differences in heat tolerance, shared DEGs were characterized. And 344 (4.6%) common DEGs were found in all groups (Fig. 4C).

### The functional annotation of DEGs

**Figure 5:**
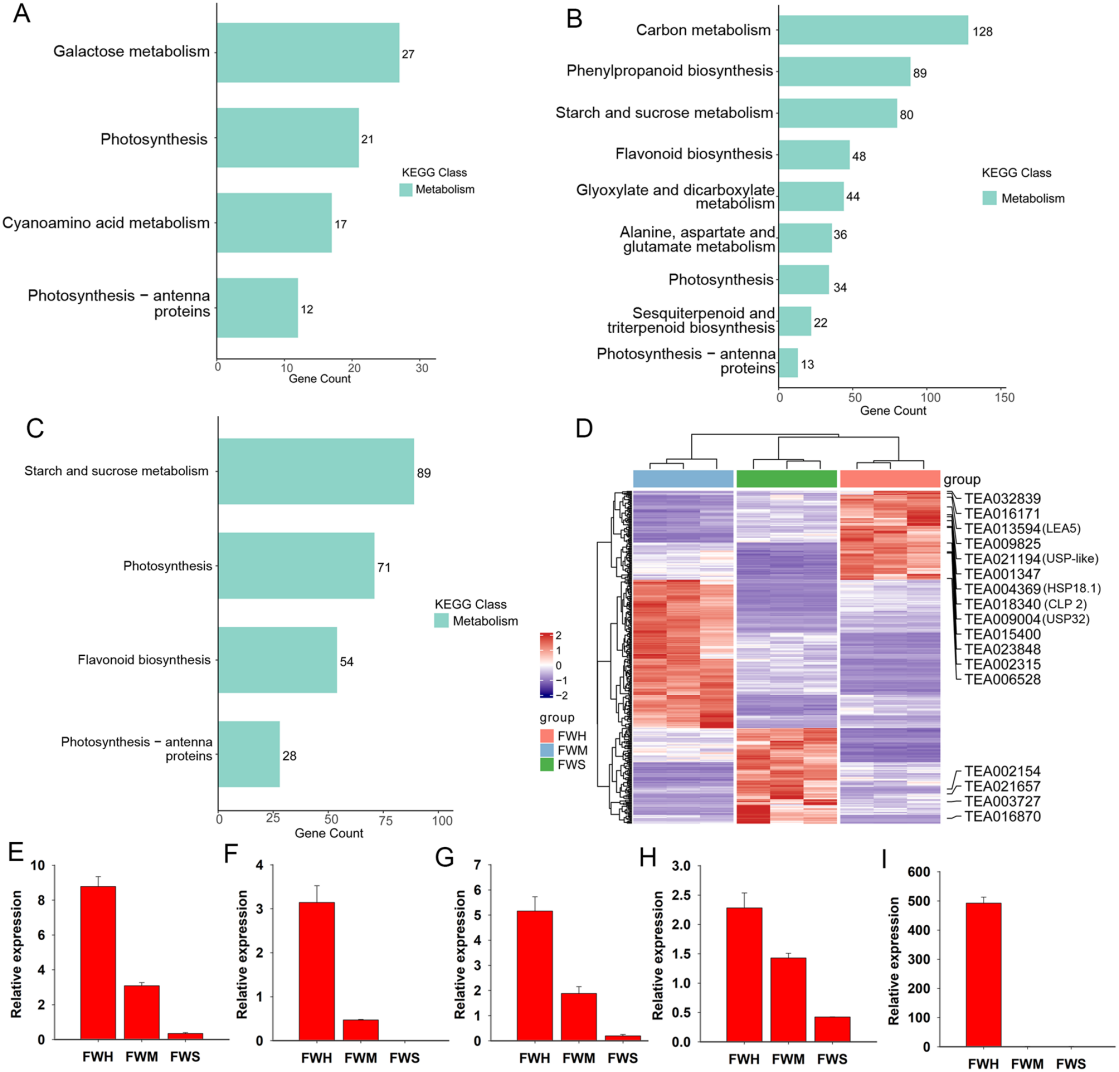
The KEGG enrichment in three groups. (**A**–**C**) Venn diagram of DEGs in FWH_vs_FWM, FWH_vs_FWS, and FWM_vs_FWS groups; (**D**) Hierarchical clustering of the 344 DEGs shared in all samples; (**E**–**I**) The RT-qPCR verification of TEA013594 (LEA5), TEA021194 (USP-like), TEA004369 (HSP18.1), TEA018340 (CLP2), and TEA009004 (USP32). Figure source: www.kegg.jp/kegg/kegg1.html.

The DEGs among different groups were further used to perform KEGG analyses against the annotated canonical pathways. KEGG analyses suggested that 4, 9, and 4 main metabolic pathways were significantly enriched in the FWH_vs_FWM, FWH_vs_FWS, and FWM_vs_FWS groups, respectively (Fig. 5A–C). These pathways included galactose metabolism, photosynthesis, and cyanoamino acid metabolism in FWH_vs_FWM (Fig. 5A). In the FWH_vs_FWS groups, the majority of pathways were enriched into energy synthesis and metabolism, including carbon metabolism, starch and sucrose metabolism, and photosynthesis. In addition, amino acid metabolism and flavonoid biosynthesis pathways were also enriched (Fig. 5B). Notably, energy-related pathways were significantly enriched in FWH_vs_FWM, including starch and sucrose metabolism and photosynthesis (Fig. 5C). In summary, photosynthesis was the key pathway influenced by heat stress among the three comparison groups. We further analyzed the 344 common DEGs that were screened in all groups and found that the genes encoding molecular chaperone were significantly up-regulated in FWH, a heat-resistant variety, such as *universal stress protein PHOS32* (*USP32*), *Chaperonin-like RbcX protein 2* (*CLP2*), *universal stress protein A-like protein* (*USP-like*), *18.1 kDa class I heat shock protein* (*HSP18.1*), and *late embryogenesis abundant protein* (*LEA5*) (Fig. 5D), and all genes were verified by RT-qPCR assay (Fig. 5E–I). These genes potentially contributed to the heat resistance in ‘FWH’ variety under long-term heat stress.

### Pathway enrichment analysis combining transcriptome with metabolome

**Figure 6:**
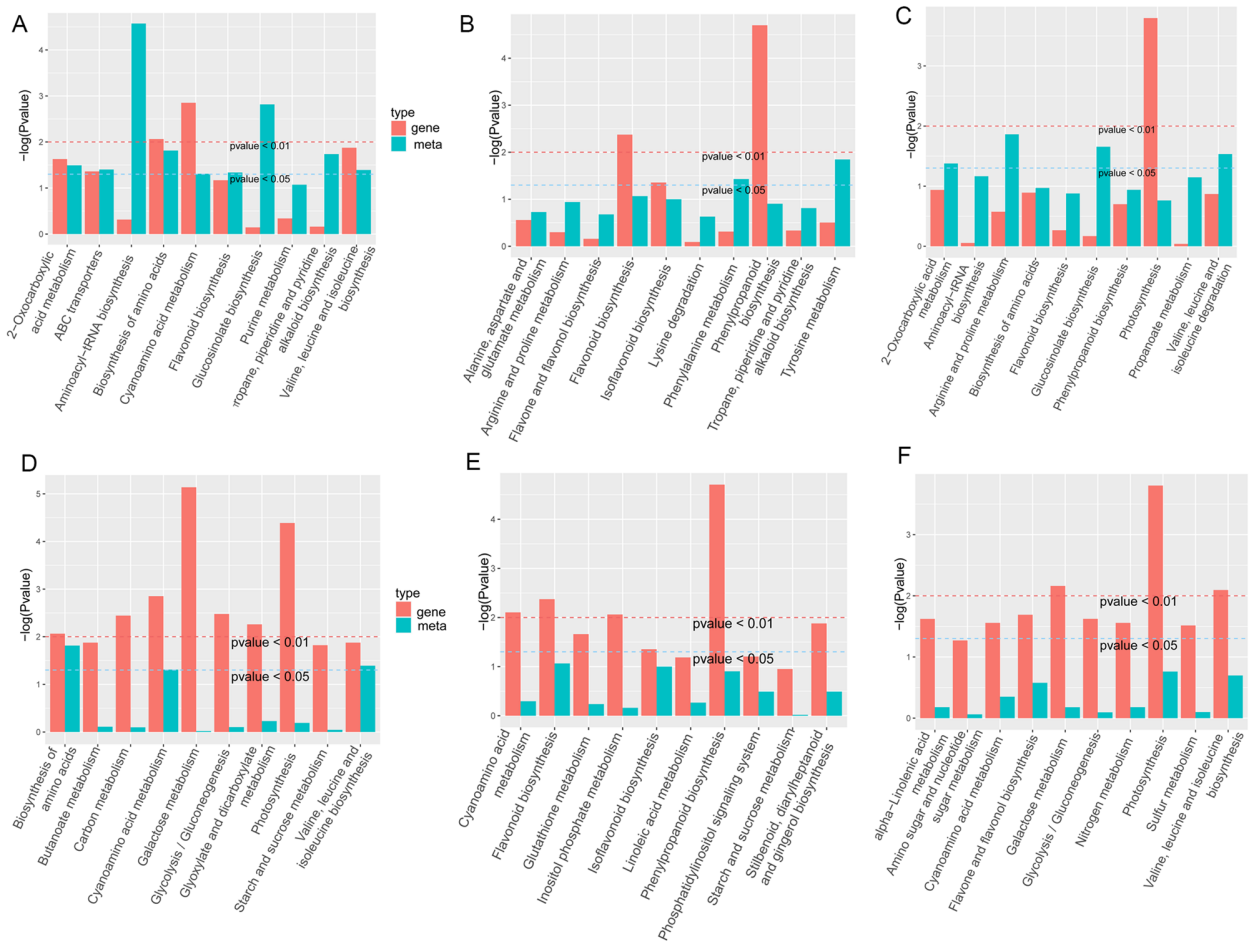
The top ten KEGG pathways combined with the metabolome and transcriptome. (**A**–**C**) The top ten KEGG pathways of FWH_vs_FWM, FWH_vs_FWS, and FWM_vs_FWS based on metabolome; (**D**–**F**) The top ten KEGG pathways of FWH_vs_FWM, FWH_vs_FWS, and FWM_vs_FWS based on transcriptome. Figure source: www.kegg.jp/kegg/kegg1.html.

In order to integrally investigate the metabolic pathways and gene expression under heat stress, transcriptome and metabolome enrichment was conducted by combining analysis. As shown in Fig. 6, the top ten KEGG pathways were enriched based on metabolome, and the results suggested that these pathways, including aminoacyl—tRNA biosynthesis, glucosinolate biosynthesis, biosynthesis amino acids, and alkaloid biosynthesis, were significantly enriched in the FWH_vs_FWM group. In the FWH_vs_FWS group, amino acid metabolisms such as tyrosine metabolism, phenylalanine metabolism, arginine and proline metabolism, and alanine, aspartate and glutamate metabolism were preferentially enriched (Fig. 6B). A similar pathway-enriched pattern was also found in the FWM_vs_FWS group (Fig. 6C). In addition, these pathways associated with phenylpropanoid biosynthesis, flavonoid biosynthesis, and photosynthesis were also enriched in all groups (Fig. 6A–C). Based on transcriptomic enriched results, these genes associated with photosynthesis, amino acid metabolism, and phenylpropanoid and flavonoid biosynthesis were significantly expressed in the FWH_vs_FWM, FWH_vs_FWS, and FWM_vs_FWS groups (Fig. 6D–F). According to these results, we found that the pathways affected by heat stress were mainly concentrated in amino acid metabolism, photosynthesis, phenylpropanoid and flavonoid biosynthesis.

### Expression of the genes on flavonoids biosynthesis

**Figure 7:**
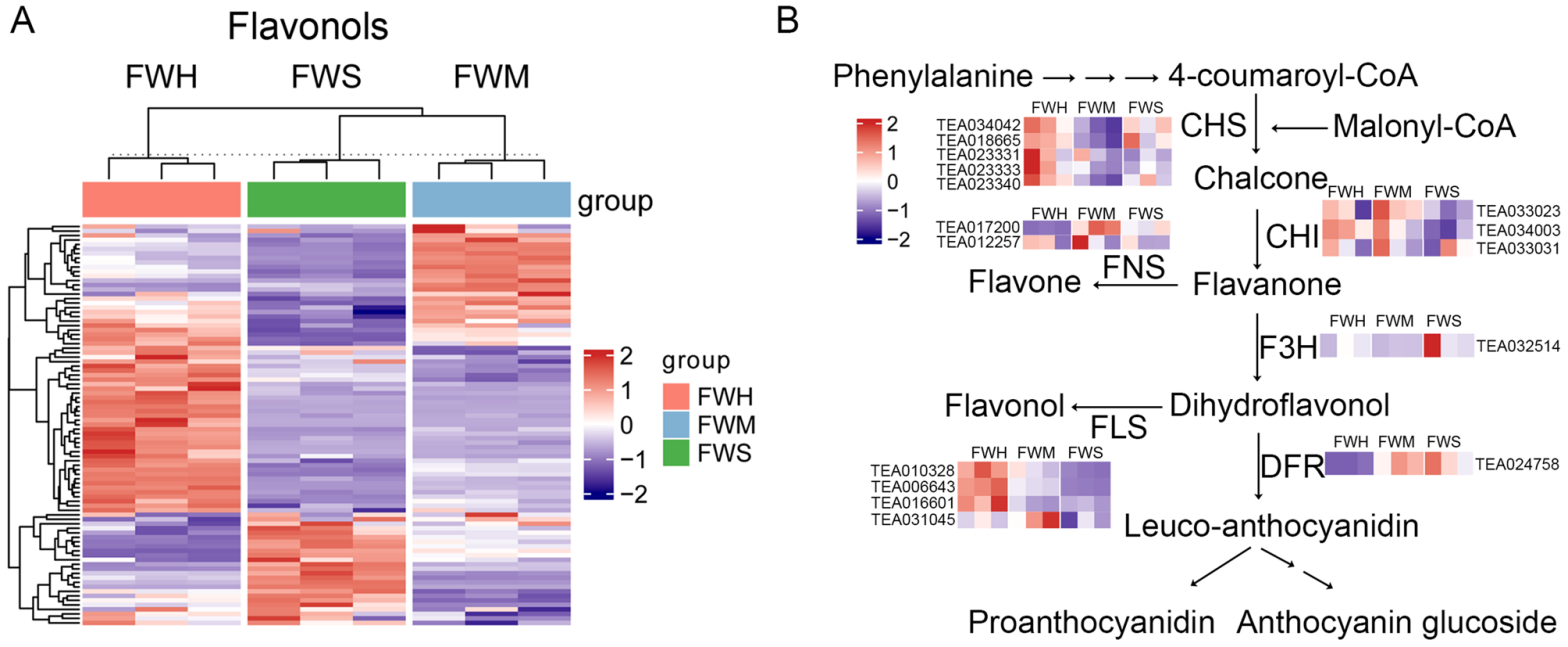
Identification of DEGs involved in flavonoids biosynthesis. (**A**) The heat map of differentially accumulated flavinols in ‘FWH’, ‘FWM’ and ‘FWS’ varieties; (**B**) the DEGs in flavonoids biosynthesis pathway. *CHS* chalcone synthase, *CHI* chalcone flavanone isomerase, *F3H* flavanone 3P-hydroxylase, *DFR* dihydroflavonol 4-reductase, *FLS* flavonol synthase, *FNS* flavone synthase.

Based on the above analyses, the pathway associated with flavonoid biosynthesis was obviously enriched during heat stress (Figs. 3, 6), which has caught our attention. In addition, we hypothesize that the drastic accumulation of H_2_O_2_ contributed to the deterioration of heat-tolerance in the ‘FWS’ variety. Previous findings indicated that, except for antioxidant enzymes, flavonoids may serve as ‘secondary’ antioxidant system that is activated as a consequence of the diminishing of antioxidant enzyme activity^29^. In this work, such flavonoids, including anthocyanins, flavones, flavonols, flavanols, and flavanones, were characterized (Supplementary Table S1). Interestingly, the majority of flavonols were remarkably up-regulated accumulation in heat-tolerant ‘FWH’ and ‘FWM’ varieties (Fig. 7A and Supplementary Fig. S1). In order to investigate what genes contributed to the accumulation difference of flavonols among the three groups, the key synthetase genes involved in flavonoids biosynthesis were identified (Supplementary Table S6). According to the results, three flavonol-related synthetase genes were differentially expressed. The *CHS* (TEA023331, TEA023333, and TEA023340) in the early pathway was highly expressed in the ‘FWH’ and ‘FWM’ varieties (Fig. 7B). Two *CHI* (TEA033023 and TEA034003) genes were significantly down-regulated in the ‘FWS’ (Fig. 7B). Notably, three flavonol synthase genes (*FLS*, TEA010328, TEA006643 and TEA016601) was significantly up-regulated in ‘FWH’ varieties (Fig. 7B). These results implied that the biased expression of the *CHS*, *CHI*, and *FLS* collaboratively results in abundant accumulation of flavonol in ‘FWH’ varieties.

## Discussion

In most tea-growing regions, extremely high temperature is tightly associated with a subsequent decline in tea yield and quality with an increase in tea plant wither and fall^30,31^. The development of improved heat-adaptive tea varieties is a key target for breeders. Therefore, in our previous work, a heat-sensitive variety ‘Wuniuzao’ and a heat-tolerant variety ‘Fudingdabaicha’ were used as parents to generate the F1 hybrid. Among these F1 hybrids, ‘FWH’ showed a remarkable heat stress tolerance phenotype, and the leaves of ‘FWM’ were partly injured, while the leaves of ‘FWS’ were severely deteriorated under high temperature (Fig. 1A–C). In the current study, comparative physiological analysis, metabonomics, and transcriptomics were remarkably used to investigate the metabolite accumulation pattern differences and uncover the key molecular cues contributing to the heat stress tolerance of tea varieties. To comprehensively understand the response to heat stress, a physiological analysis of three F1 tea varieties suggested a differential phenotypic response to heat stress, potentially due to the genetic diversity of F1 generation^32^. In this study, high temperature causes oxidative damage of membrane system, potentially leading to a decrease in photochemical efficiency, and the leaves of the heat-sensitive variety ‘FWS’ were found to be severely influenced by increasing of MDA content (Fig. 1E)^33^. Previous findings suggested that high proline accumulation in heat stress condition ensures faster recovery from heat damage in plants and excessive proline accumulation exhibits better adaptation to abiotic stresses^34,35^. Therefore, proline up-accumulation confers the tolerance of ‘FWH’ to heat stress (Fig. 1F). Furthermore, it has been suggested that heat stress induces the production of ROS in plants, and over-accumulation of ROS has deleterious impacts on plants by causing oxidative lesions to biomacromolecules and cell membranes^36^. Our results also found the H_2_O_2_ and O_2_^−^ content were significantly up-accumulated in the heat-sensitive ‘FWS’ variety, ultimately, causing antioxidative damage in leaves. In addition, the antioxidant activity such as SOD, POD, and CAT was also decreased in ‘FWS’. These results indicated that ‘FWH’ exhibits a better ability to withstand heat stress via the intrinsic antioxidant system and vice versa in ‘FWS’. According to the results in herbaceous plants, such morphologic changes, including mesophyll thickness decreasing, spongy tissue loosing, and palisade cell disordering, were observed under heat stress treatments^13^, but the differences of anatomical characteristics among the three varieties were observed (Fig. 1M–O).

It is reported that the photosynthesis efficiency of the tea plant with the C3 photosynthetic mechanism is the most efficient at an optimum temperature of 25–30 °C^37,38^. Photosynthesis is one of the processes influenced by high-temperature in plants, and ambient temperature is a limiting factor determining the CO_2_ fixation capacity and the activity of the photosynthetic apparatus^39^. According to the records, extreme high-temperature weather, above 35 °C, has frequently occurred in many tea-production areas in China^40^. In this study, higher temperature has been heavily impaired the leaves in heat-sensitive varieties consecutively inhibiting the photosynthetic mechanism. Transcriptome analysis found that the differentially expressed genes involved in photosynthesis and carbon metabolism were significantly enriched among different groups, indicating high-temperature stress has an effect on photosynthesis in tea plants (Fig. 5A–C).

One of the plant heat-tolerance mechanisms is that compatible N-rich solutes were induced, such as free amino acids, that confer heat tolerance because they act as osmolytes to regulate ion transport, stomatal opening, and enzyme activity^41^. Previous findings have shown that the proline content was significantly accumulated in poplar under heat stress^42^. Besides being fundamental substances for protein synthesis, many amino acid such as lysine, methionine, and polyamines generated either from arginine or ornithine, turned out to have active roles in plant responses to environmental stresses^41^. In this study, proline accumulated significantly in heat-tolerant ‘FWH’ variety exposed to high temperature (Fig. 1F), and amino acid metabolism pathways such as arginine and ornithine metabolism, proline metabolism and alanine, and aspartate and glutamate metabolism were frequently enriched based on metabolomic analysis (Fig. 3). In celery, the amino acid accumulation pattern and type were influenced by heat stress, and the biosynthesis of arginine and serine were enhanced^13^. These results suggested that the synthesis of amino acids potentially acts as a common response to heat stress in plants^41^.

According to morphological and physiological investigations, genome-wide transcriptional analysis further contributes to understand the molecular programming and mechanism of heat stress in tea plants. Molecular chaperones play an important role in plant responses to heat stress. In this study, we found that some genes encoding molecular chaperone proteins such as *USP32*, *CLP2*, *USP-like*, *HSP18.1*, and *LEA5*, were significantly up-regulated in heat-tolerant tea varieties. It has been proven that HSPs and HSFs synergistically mediate regulation of heat-related gene expression, conferring thermotolerance in tea plant^23,43^. The HSF network in plants is regulated at the transcriptional level by the cooperation of different HSF members and by interactions with HSP chaperones such as HSP90 and HSP70^23^. In addition, the small molecular chaperon plays a role in responding to heat stress. Up-regulation expression of *CsHSP17.6* was found to stabilize chloroplast membrane protein dynamics by ROS scavenging in tea plant^26^. In addition, under high temperature, *CsHSP24.6* and its target gene, *CspTAC5*, interacted to enhance the heat tolerance in tea plant^25^. In the current study, a small heat shock protein (*HSP18.1*) was found to be significantly up-regulated in the heat-tolerant ‘FWH’ variety (Fig. 5D). In *Arabidopsis*, overexpression of chloroplastic *sHSP26* enhanced substantial heat-tolerance under a continuous high temperature regimen and further improved the accumulation of photosynthetic pigments, biomass, and seed yield^44^. Furthermore, such proteins called universal stress proteins (USPs), which are first identified in prokaryotes and were widespread and conserved in flowering plants, were preferentially expressed in the ‘FWH’ variety during heat stress (Fig. 5D)^27^. USPs are a small stress-inducible protein with an estimated molecular weight of 13.5 kDa and located in the cytosolic fraction. For example, 44 proteins orthologous with the USP from *Escherichia coli* were found in *Arabidopsis*^45^. Previous findings indicated that AtUSP acting as a protein chaperone prevents denaturation of intracellular core macromolecules from heat shock or oxidative stress^28^. HRU1, a low-oxygen-responsive USP induced by anoxic conditions, served as a chaperone that regulated H_2_O_2_ production via targeting GTPase ROP2 and the NADPH oxidase RbohD in *Arabidopsis*^46^. In *Sulfolobus acidocaldarius*, SaUspA acts as a molecule partner to stimulate the activity of the phosphatase PP2A at salinity stress^47^. In the current study, two USP genes (USP32 and USP-like) were up-regulated in the ‘FWH’ variety, indicating that the USP genes served as late response genes and contributed to the heat tolerance of the tea variety during long-term heat stress.

Flavonoids were proven to provide protection against abiotic stress in plant by acting as scavengers for ROSs, which is the consequence of stress^48^. In tea plants, excessive accumulation of flavonoid content play a role in improving the tolerance of plants to drought^49^. In our results, ROSs such as H_2_O_2_ and O_2_^−^ content were barely accumulated in the heat-tolerant ‘FWH’ variety, partly because of antioxidant activity (Fig. 1), and the excessive accumulation of flavonols, such as kaempferol-3-arabinopyranoside, kaempferol-4′-O-β-d-glucopyranoside, and quercetin-3-O-(6″-*O*-acetyl)-galactoside, potentially contributed to the elimination of ROSs (Fig. 7A and Supplementary Table S1). Flavonols, in fact, were found to directly modulate ROS homeostasis during high-temperature stress in order to control pollen tube growth and integrity^50^. In soybean, the accumulation of endogenous flavonoids alleviated heat-induced ROS damage^51^. In addition, the up-regulation of *FLS* could potentially serve as a key factor responsible for flavonol biosynthesis (Fig. 7B).

## Materials and methods

### Plant materials

Three tea varieties, which are the F1 progenies generated from a heat-sensitive variety ‘Wuniuzao (*Camellia sinensis* var. sinensis)’ and a heat-tolerant variety ‘Fudingdabaicha (*C. sinensis* var. *sinensis*)’, named ‘FWH’, ‘FWM’, and ‘FWS’ in this work, were used as materials to perform widely targeted metabolomic analyses, determination of metabolite content, and transcriptome sequencing. All varieties were planted in the same tea plantation and were grown under natural environmental conditions, and typical phenotypes under heat stress were described in Results. Here, the phenotypic observation was conducted during June 1–August 22, 2022. And the average high temperature is 34.7 °C, the average low temperature is 24.3 °C, and the temperature exceeds 35 °C for 49 days in tea plantation. After extreme heat stress, young buds with two leaves from every variety were sampled and frequently frozen in liquid nitrogen. All samples were stored at − 80 °C until further analysis.

The tea plants used in our research, including the parents and offspring, are all sourced from the National Small and Medium Leaf Tea Germplasm Resources Nursery (Changsha). The three F1 hybrid offspring involved in this study were obtained through hybridization. All the materials used in this study comply with the IUCN Policy Statement on Research Involving Species at Risk of Extinction and the Convention on the Trade in Endangered Species of Wild Fauna and Flora.

### Paraffin sections preparation and observation

The young leaves of tea varieties were cut into pieces of 3 mm × 5 mm, and immediately immersed into a plant fixative FAA solution (10% formaldehyde, 50% alcohol, 5% acetic acid, and 35% water) for 24 h. And the samples were dehydrated by gradient alcohol (70%, 90%, 95%, 100%, and 60 min for each step). After discardding alcohol, the samples were embedded in paraffin, respectively. Serial sections were cut using a Leica microtome (Leica, Japan) and further stained by SafraninO-Fast Green Staining (LabGene, Shanghai, China). After sealing with neutral gum, the permanent slides were observed and subsequently photographed using a microscope (Zeiss, Germany).

### Determination of enzyme activities, and metabolites content

The SOD (U/g FW) activity was measured according to previous descriptions^52^. Briefly, 0.1 g sample was weighted and ground. Added 1 mL 0.5 mol/L PBS buffer (pH 7.8) and centrifuged at 10,000 × g for 10 min at 4 °C. Transferred the supernatant into a new tube. Then, 45 µL of 100 µM EDTA, 100 µL of 750 µM nitroblue tetrazolium solution, 3 µL of xanthine oxidase, 18 µL of the sample, and 35 µL of 130 mM methionine solution were orderly added into 96-well plates. The control tube included 18 µL of double-distilled water instead of the crude. After mixing, the samples were incubated for 30 min at room temperature, and absorbance was determined at 560 nm. SOD enzyme activity was calculated based on fresh weight. At a percentage inhibition in the above xanthine oxidase conjugate reaction system of 50%, SOD enzyme activity in the reaction system was defined as unit enzyme activity.

The CAT (U/g FW), PPO (U/g FW) and POD (U/g FW) activities were determined using kits (Solarbio Life Science, Beijing, China). And the content of H_2_O_2_ (μmol/g FW), and O_2_^−^ (nmol/g FW) was determined by using kits (Solarbio Life Science, Beijing, China). All measurements were conducted according to the manufacturer’s descriptions^53^.

The content of flavone was determined using the NaNO_2_-AlCl_3_–NaOH method^54^. 0.2 g sample was ground and homogenized using 2 mL of pre-cold 80% methanol and was incubated for 30 min in ultrasonic bath. The solution was centrifuged at 10,000*g* at 25 °C for 10 min and the supernatant was collected. The supernatant was combined and standardized to a final volume of 10 mL using 80% methanol. The spectrophotometer microplate reader was preheated for 30 min and the absorbance was measured at the wavelength of 510 nm. The content of MDA was determined according to previous descriptions^55^. Simply, 0.2 g sample was ground and homogenized in 5 mL of 5% (w·v^−1^) trichloroacetic acid. The homogenates were centrifuged at 3000*g* for 10 min. 2 mL of the supernatant was mixed with 2 mL of 10% (w·v^−1^) thiobarbituric acid. The mixtures were incubated in boiling water baths for 10 min, cooled to room temperature, and then centrifuged at 3000*g* for 30 min. The absorbances of the supernatants were measured at 450, 532, and 600 nm. The content of Pro (µg g^−1^ FW) was measured by the biochemical kits (Solarbio Science, Beijing, China) according to the manufacturer’s descriptions.

### Metabolite extraction and metabolomic analysis

Metabolite extraction was performed according to previous methods and modified^56^. 100 mg freeze-dried leaves were smashed, homogenized with 1.0 mL of 70% aqueous methanol and extracted overnight at 4 °C. After centrifuged at 10,000 × *g* for 10 min at 4 °C, the extracts were absorbed on a CNWBOND Carbon-GCB SPE Cartridge (250 mg, 3 mL; ANPEL, Shanghai, China) and filtered (SCAA-104, 0.22 μm pore size; ANPEL, Shanghai, China) for LC–MS analysis. LC–ESI–MS/MS system (HPLC, Shim-pack UFLC SHIMADZU CBM30A system, www.shimadzu.com.cn/; MS, Applied Biosystems 4500 Q TRAP, http://www.appliedbiosystems.com.cn/) was used to analyze sample extracts. The liquid chromatography analytical conditions were based on the previous description^57^.

Linear ion trap (LIT) and triple quadrupole (QQQ) scans were acquired on a triple quadrupole-linear ion trap mass spectrometer (API 4500 Q TRAP LC/MS/MS System; Boston, USA) equipped with an ESI Turbo Ion Spray interface, operating in both positive and negative ion modes and controlled by Analyst 1.6.3 software (AB Sciex, Singapore). The ESI source operation parameters were as follows: ion source, turbo spray; source temperature, 550 °C; ion spray voltage (IS), 5500 V; and ion source gas I (GSI), gas II (GSII), curtain gas (CUR) set to 55, 60 and 25 psi, respectively. The collision activated dissociation (CAD) was set at “high”. Instrument tuning and mass calibration were performed with 10 and 100 μM polypropylene glycol solutions in QQQ and LIT modes, respectively. QQQ scans were acquired via multiple reaction monitoring (MRM) experiments with col-lision gas (nitrogen) set to 5 psi. De-clustering potential (DP) and collision energy (CE) were optimized for individual MRM transitions. A specific set of MRM transitions was monitored for each period according to the metabolites eluted within the period^55^.

### Mass spectrometry data and statistical analyses

Mass spectrometry (MS) data acquisition and processing were performed as described previously^58,59^. The analyses of the primary and secondary MS data were conducted based on the self-built database MWDB (Metware Biotechnology Co., Ltd. Wuhan, China). Metabolite quantification was accomplished with data acquired in MRM mode by QQQ-MS^60^. Metabolites with a fold change ≥ 2 or a fold change ≤ 0.5 were identified as upregulated or downregulated^56^.

### Library preparation for RNA sequencing

The RNA sequencing (RNA-seq) libraries were constructed using NEBNext^®^ Ul-traTM RNA Library Prep Kit for Illumina^®^ (NEB, USA) following the manufacturer’s descriptions. Briefly, messenger RNA (mRNA) was purified from total RNA using poly-T oligo attached magnetic beads. Fragmentation was performed using divalent cations under elevated temperature in NEBNext First Strand Synthesis Reaction Buffer (5×). First strand cDNA was synthesized using random hexamer primer and M-MuLV Reverse Transcrip-tase (RNase H-). Second strand cDNA synthesis was subsequently performed using DNA Polymerase I and the RNA was digested by RNase H. Remaining overhangs were con-verted into blunt ends. After adenylation of 3′ ends of DNA fragments, NEBNext Adaptor with hairpin loop structure were ligated to prepare for hybridization. In order to select cDNA fragments of preferentially 250 ~ 300 bp in length, the library fragments were purified with AMPure XP system (Beckman Coulter, Beverly, USA). Then 3 µL USER Enzyme (NEB, USA) was used with size-selected, adaptor-ligated cDNA at 37 °C for 15 min fol-lowed by 5 min at 95 °C before PCR. Then PCR was performed with Phusion High-Fidelity DNA polymerase, Universal PCR primers and Index (X) Primer. At last, PCR products were purified (AMPure XP system) and library quality was assessed on the Agilent Bio-analyzer 2100 system. The library preparations were sequenced on an Illumi-na^®^HiSeq2500 platform and 125 bp/150 bp paired-end reads were generated.

### Function annotation and expression analysis

The raw data were filtered by removing low quality reads and adaptors and were changed into clean reads. The HISAT v2.1.0 package was used to construct the index and mapped clean reads to the NCBI GeneBank. The feature Counts v1.6.2 package was used to count the gene alignment, and then calculate the Fragments Per Kilobase per Million (FPKM) of each gene based on the gene length. The DESeq2 v1.22.1 was used to analyze the differential expression between the two groups, and the P value was corrected using the Benjamini and Hochberg method. Gene function was annotated according to the tea plants databases (TPIA, http://tpia.teaplants.cn/).

### Real-time quantitative PCR (RT-qPCR) analysis

Total RNA was extracted using a RNAprep pure Tissue Kit (TIENGAN, China). The first strand of the reverse transcribed cDNA was synthesized using the specifications of the Monad first-strand cDNA Synthesis Kit (Monad, China). The primers were designed using NCBI’s primer designing tool, PRIMER-BLAST (Supplementary Table S7). The ABI7500 quantitative PCR instrument was adopted to perform real-time fluorescence quantitative PCR. All data were obtained from three biological repetitions and experiments were repeated three times.

### Statement of experimental methods

We confirm that all methods were performed in accordance with the relevant guidelines/regulations/legislation.

## Conclusion

In this work, we compared the physiological and transcriptional variation among different heat-tolerant tea plant varieties. We found that the H_2_O_2_ content is significantly increased in the heat-sensitive variety, with activity decreasing of scavenging enzymes. Metabonomic analysis found that multiple pathways including amino acids metabolism, photosynthesis, phenylpropanoid and flavonoid biosynthesis were significantly influenced under heat stress. In addition, transcriptomic results suggested that molecular chaperons and chaperon-like proteins, flavonols accumulation collaboratively play positive roles in heat-tolerance of tea plant under ambient high temperature.

### Supplementary Information

Supplementary Figure S1.

Supplementary Table S1.

Supplementary Table S2.

Supplementary Table S3.

Supplementary Table S4.

Supplementary Table S5.

Supplementary Table S6.

Supplementary Table S7.

## Data Availability

Data presented in this study is deposited into NCBI and the accession ID is PRJNA973196. All data generated or analyzed in this work are included in this published article and its supplementary information files.
