# Supplementary figures and images for: Investigation of heat stress responses and adaptation mechanisms by integrative metabolome and transcriptome analysis in tea plants (Camellia sinensis)
Source: Sci Rep. 2024 May 1;14:10023. doi: 10.1038/s41598-024-60411-0 (PMC11063163; doi:10.1038/s41598-024-60411-0)

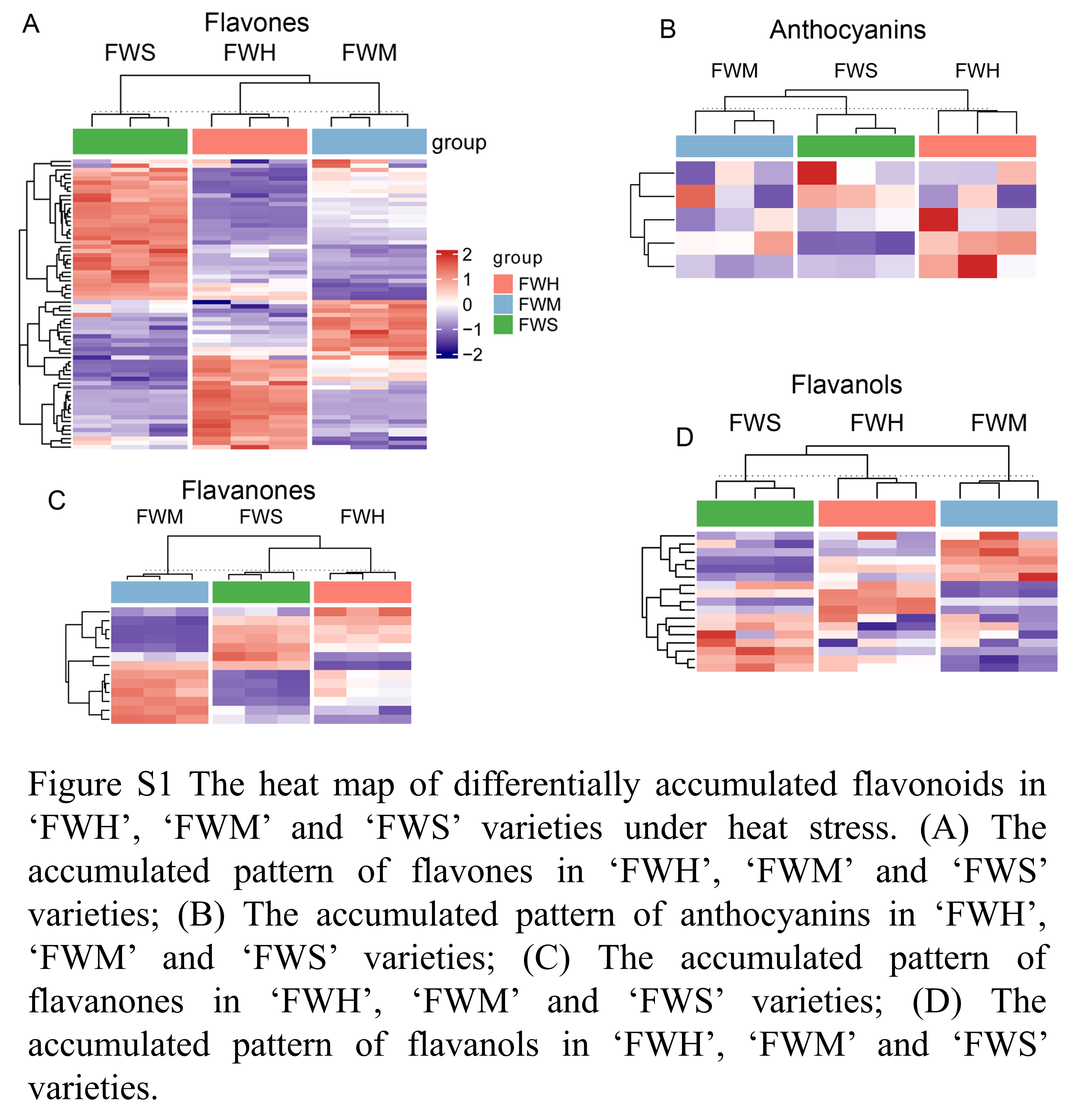

Supplement: Supplementary file 1 — Supplementary Figure S1. [file 41598_2024_60411_MOESM1_ESM.tif]
